# Prognostic significance of a signature based on senescence-related genes in colorectal cancer

**DOI:** 10.1007/s11357-024-01164-6

**Published:** 2024-04-25

**Authors:** Zoltan Ungvari, Anna Ungvari, Giampaolo Bianchini, Balázs Győrffy

**Affiliations:** 1 Vascular Cognitive Impairment, Neurodegeneration and Healthy Brain Aging Program, Department of Neurosurgery, University of Oklahoma Health Sciences Center, Oklahoma City, OK USA; 2 Stephenson Cancer Center, University of Oklahoma, Oklahoma City, OK USA; 3 Oklahoma Center for Geroscience and Healthy Brain Aging, University of Oklahoma Health Sciences Center, Oklahoma City, OK USA; 4 Department of Health Promotion Sciences, College of Public Health, University of Oklahoma Health Sciences Center, Oklahoma City, OK USA; 5 International Training Program in Geroscience, Doctoral College/Department of Public Health, Semmelweis University, Budapest, Hungary; 6 Department of Public Health, Semmelweis University, Semmelweis University, Budapest, Hungary; 7 Department of Medical Oncology, IRCCS Ospedale San Raffaele, Milan, Italy; 8 Dept. of Bioinformatics, Semmelweis University, 1094 Budapest, Hungary; 9 Dept. of Biophysics, Medical School, University of Pecs, 7624 Pecs, Hungary; 10 Cancer Biomarker Research Group, Institute of Molecular Life Sciences, HUN-REN Research Centre for Natural Sciences, 1117 Budapest, Hungary

**Keywords:** Aging, Gerooncology, Cancer, Senescence, Colorectal cancer

## Abstract

Colorectal cancer, recognized as a quintessential age-related disease, underscores the intricate interplay between aging mechanisms and disease pathogenesis. Cellular senescence, a DNA damage-induced cellular stress response, is characterized by cell cycle arrest, the expression of an inflammatory senescence-associated secretory phenotype, and alterations in extracellular matrix metabolism. It is widely recognized as a fundamental and evolutionarily conserved mechanism of aging. Guided by geroscience principles, which assert that the pathogenesis of age-related diseases involves cellular mechanisms of aging, this study delves into the role of senescence-related genes in colon cancer progression. Leveraging a gene set reflective of senescence-associated pathways, we employed uni- and multivariate Cox proportional hazards survival analysis combined with the determination of the false discovery rate to analyze correlations between gene expression and survival. The integrated database of 1130 colon cancer specimens with available relapse-free survival time and relapse event data from ten independent cohorts provided a robust platform for survival analyses. We identified senescence-related genes associated with differential expression levels linked to shorter survival. Our findings unveil a prognostic signature utilizing cellular senescence-related genes (hazard ratio: 2.73, 95% CI 2.12–3.52, *p* = 6.4E − 16), offering valuable insights into survival prediction in colon cancer. Multivariate analysis underscored the independence of the senescence-related signature from available epidemiological and pathological variables. This study highlights the potential of senescence-related genes as prognostic biomarkers. Overall, our results underscore the pivotal role of cellular senescence, a fundamental mechanism of aging, in colon cancer progression.

**Supplementary Information:**

The online version contains supplementary material available at 10.1007/s11357-024-01164-6.

## Introduction

Colorectal cancer represents a significant public health challenge, accounting for a substantial portion of cancer-related morbidity and mortality globally [1–5]. In the United States and European Union, colorectal cancer ranks among the most prevalent malignancies, with incidence rates steadily rising over recent decades [1–5]. Colorectal cancer exerts a profound impact on affected individuals, their families, and healthcare systems, underscoring the urgent need for comprehensive research efforts aimed at elucidating its underlying mechanisms and improving clinical outcomes [2].

Colorectal cancer is recognized as a quintessential age-related disease, with its incidence escalating with advancing age [1–7]. As individuals age, they become increasingly susceptible to the development and progression of this malignancy [1, 6, 7]. In this context, the burgeoning field of geroscience emerges as a valuable framework for comprehending the pathogenesis of age-related diseases, including colorectal cancer [1, 6–8]. By elucidating the role of fundamental molecular and cellular processes of aging, geroscience offers new insights into the origins and progression of colorectal cancer. In the context of colorectal cancer research, the application of geroscience principles hold promise for uncovering novel therapeutic targets and prognostic and diagnostic biomarkers, ultimately improving patient outcomes and quality of life.

Cellular senescence, characterized by chronic DNA damage-induced cellular stress response, represents a hallmark of aging [9–13]. This evolutionarily conserved cellular mechanism of aging involves a complex array of cellular changes, including cell cycle arrest, the expression of an inflammatory senescence-associated secretory phenotype (SASP), and alterations in extracellular matrix metabolism [14–18]. Such changes contribute to the aging process and play a crucial role in the pathogenesis of an array of age-related diseases, including cancer [14–16]. Given the pivotal role of cellular senescence in aging and disease, there is a compelling rationale for studying senescence-related genes in the context of colon cancer. Understanding the prognostic significance of these genes may provide valuable insights into disease progression and guide the development of targeted therapeutic interventions.

This study was designed to investigate the prognostic significance of senescence-related genes in colon cancer, with the overarching goal of contributing to our understanding of disease progression and clinical outcomes. By leveraging a comprehensive gene set reflective of senescence-associated pathways [9], we sought to elucidate correlations between gene expression and survival outcomes in a large cohort of colon cancer samples. To achieve our research objectives, we utilized an integrated colorectal patient cohort from the Kaplan–Meier Plotter platform [19, 20]. This approach allowed for the identification of senescence-related genes associated with differential expression levels linked to survival outcomes.

## Methods

### Identifying cohorts of colon cancer patients

To perform a survival analysis in colon cancer, we looked first for colon cancer samples in two online databases: the NCBI Gene Expression Omnibus (https://www.ncbi.nlm.nih.gov/geo/) and the Genomic Data Commons Data Portal (https://portal.gdc.cancer.gov/). We only included samples with detailed genetic information (transcriptome data). To ensure consistent and reliable data, we searched for tumor samples analyzed using specific in-situ oligonucleotide array platforms. For this, we chose three platforms (GPL96, GPL571, and GPL570) that use the same probes to measure gene expression levels, reducing potential errors from using different technologies.

### Data cleaning and gene selection

The raw gene expression data from the gene arrays went through a two-step cleaning process. First, MAS5 normalization was applied, and then a scaling normalization was done to set the average expression level to 1000 for each array. To ensure consistency and avoid biases due to different chip designs, only probes present in the GPL96 platform were used. This is especially important because the GPL570 arrays have many additional probes that could introduce variation. Next, to identify the most dependable probe set for each gene, the JetSet algorithm was employed. Additionally, various quality control checks were performed, including checking for background intensity, which measures the non-specific signal unrelated to gene expression; noise levels, which refers to the random fluctuations in the data; percentage of present calls, which indicates the proportion of probes that provided reliable signals; the presence of bioBCD spikes, which control elements added to the arrays to monitor the performance of the experiment and 3′/5′ ratios of GAPDH and ACTB, which are housekeeping genes, and their expression levels should be relatively stable. Examining their 3′/5′ ratios helps assess the quality of RNA used in the experiment. By following these steps, we ensured the accuracy and reliability of our gene expression data before proceeding with further analysis.

### Setting up a signature and univariate survival analysis

Gene expression analysis was conducted using the Kaplan–Meier Plotter platform [19, 20] available at https://kmplot.com/analysis/index.php?p = service&cancer = colon. This software facilitated the comprehensive analysis necessary to identify the prognostic signature discussed. To elucidate the influence of gene expression on relapse-free survival rates in colon cancer patients, we employed a Cox regression analysis. In this study, we utilized a comprehensive gene set reflective of senescence-associated pathways, sourced from the study by Saul et al. [9]. This gene set, known as SenMayo, has been validated for its enrichment in senescent cells across various tissues and species. First, each gene was investigated individually to assess its impact on patient survival, and genes were weighted according to whether the resulting HR was larger than one (1) or between zero and one (− 1). The integrated senescence-derived signature was determined as the average weighted expression of all genes. The complete list of all genes with probe sets and weights is provided in Supplemental Table 1**.**

In the survival analysis, to avoid missing important information due to choosing a specific cut-off point for gene expression, we tested all possible values between the lowest and highest quartiles of expression levels for the combined signature. However, the large number of tests conducted can lead to false positive results. To account for this, we computed the False Discovery Rate (FDR) to correct for multiple hypothesis testing. Furthermore, we also conducted separate survival analyses using each of the included datasets, independently of the analysis of the entire cohort.

To visually represent the differences in survival based on gene expression, we generated Kaplan–Meier survival plots. The plots utilize the cut-off points identified in the univariate analysis to illustrate how gene expression levels impacted patient survival rates.

### Multivariate analysis

We performed multivariate Cox regression to assess the combined effect of the gene signature and other relevant clinical and pathological features on relapse-free patient survival. The investigated clinical parameters include gender, pathological T, N, M, stage, grade, the level of microsatellite instability, and location of the tumor. Due to a large number of missing data in the clinical database, each analysis was performed in pairs (e.g., the integrated senescence-derived gene signature and gender in one setting).

## Results

### Database setup

The complete integrated database includes 1130 tumor samples with available relapse-free survival time and transcriptome-level gene expression. The mean follow-up was over four years with 24% of the patients having a relapse event. Except for relapse-free survival data, not all patients had each of the clinical parameters available. The majority of patients were male. Although only half of the patients had TNM stage available, the majority were T 3/4, N 0/1, and M 0. In the pathological stage, 2 and 3 dominate the dataset, and most of the samples with available MSI status had a disease with stable or low MSI. The detailed clinical characteristics with specific patient numbers for the entire integrated database are provided in Table 1.

**Table 1:** Clinical characteristics of the included colon cancer patients. Note that with the exception of relapse-free survival data not all patients had all data available

Feature	*n* (%)
Included datasets	10
Survival	
Mean follow-up	50 months
Number of patients with an event	271
Sex	
Female	435 (46.92%)
Male	492 (53.08%)
Pathological T	
1	11 (2.25%)
2	47 (9.61%)
3	328 (67.08%)
4	102 (20.86%)
Pathological N	
0	257 (52.88%)
1	138 (28.4%)
2	87 (17.9%)
3	4 (0.82%)
Pathological M	
0	474 (93.31%)
1	34 (6.69%)
Grade	
1	23 (12.57%)
2	136 (74.32%)
3	24 (13.11%)
Stage	
1	90 (8.69%)
2	504 (48.65%)
3	401 (38.71%)
4	41 (3.96%)
Location	
Proximal	336 (42.75%)
Distal	450 (57.25%)
Microsatellite-instability	
Stable	138 (20.63%)
Stable or low	450 (67.26%)
High	81 (12.11%)

### Univariate survival analysis

**Fig. 1:**
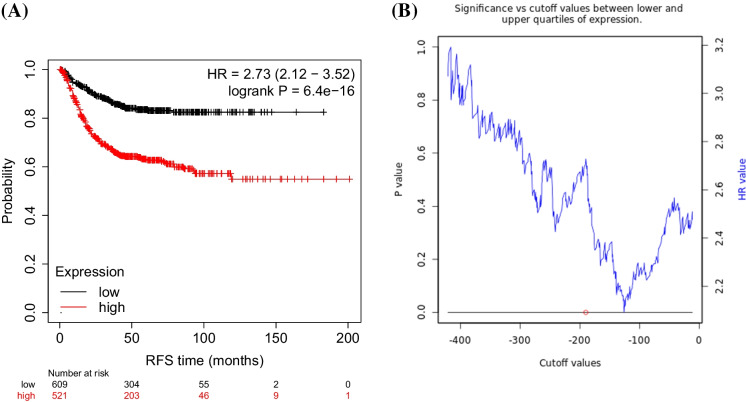
Correlation between the senescence-derived signature and survival in colon cancer. The Kaplan–Meier survival plot is based the mean weighted gene expression of the senescence-related gene signature (**A**). The significance vs cutoff plots shows that the signature remains robust regardless of the used cutoff value (**B**). The red circle marks the lowest p-value which was used when drawing the Kaplan–Meier plot. RFS = relapse-free survival, HR = hazard rate

The combined signature was established by using the mean expression of all included genes as described in the methods section. When using the entire SenMayo senescence-associated genelist-derived signature in all available patient samples, a significant correlation was observed with relapse-free survival (HR = 2.73, 95% CI = 2.12–3.52, *p* = 6.4e − 16, see Fig. 1A), and the false discovery rate was below 1%. Notably, when checking all available cutoff values between the lower and upper quartiles of the expression of the signature, each cutoff delivered a significant *p*-value with HR values ranging between 2 and 3.2. A plot displaying the significance and the HR values vs the cutoff values is provided in Fig. 1B.

### Per dataset analysis

**Fig. 2:**
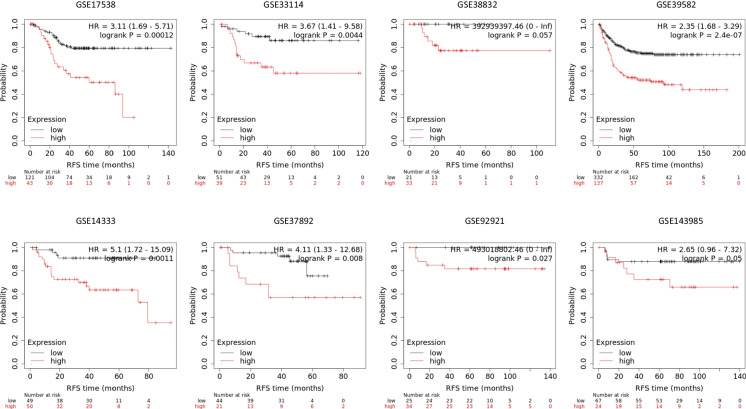
The correlation between relapse-free survival and the senescence-derived signature holds when analyzing the prognostic power in each dataset separately. Note that because not all datasets have sufficient number of cases for a separate analysis, the total sample number does not equal to the total sample number in Fig. 1. RFS = relapse-free survival, HR = hazard rate

The most robust analysis results should be reproducible in different datasets. To assess the overall prognostic power of the established signature, we separately analyzed the prognostic power in each involved dataset where a sufficient number of patients were available with follow-up data. The combined signature had a significant (*p* between 0.027 and 2.4e − 07) correlation to survival in six independent datasets and a marginal (*p* = 0.05 and *p* = 0.057) correlation in two datasets. In each of the eight investigated datasets, higher signature expression resulted in worse outcomes. The Kaplan–Meier survival plots in each dataset are provided in Fig. 2. These results suggest that the integrated signature has a robust and reproducible correlation with survival independently of the datasets used.

### Multivariate analysis

To increase the sample number available for multivariate analysis, the signature was analyzed in pairs with each investigated clinical parameter separately. The SenMayo senescence-associated genelist-derived signature retained its significance when combined with T status (*p* for signature = 9.4e − 04, *p* for T status = 3.1e − 05), N status (*p* for signature = 0.011, *p* for N status = 3.1e − 05), and stage (*p* for signature = 4.7e − 07, *p* for stage = 5.7e − 07). Sex, grade, M status, location, and microsatellite instability did not reach significance while the signature remained significant in each of the analysis settings. These results suggest that the SenMayo senescence-associated genelist-derived signature has prognostic power independent of the available clinical and pathological parameters.

## Discussion

The evaluation of senescence-related genes in colon cancer reveals their notable prognostic potential. Our study introduces a prognostic signature derived from the weighted expression of senescence-associated genes included in the SenMayo genelist, providing valuable insights into survival prediction in colon cancer. These results align with prior research, affirming the involvement of senescence in the progression of colon cancer [17, 21–23]. By intersecting geroscience and cancer biology, our study adopts a gerooncology perspective, which offers a holistic understanding of the intricate interplay between aging processes and cancer development and progression. Leveraging this perspective enhances our ability to identify novel prognostic biomarkers, ultimately improving patient outcomes in colon cancer management.

Senescent cells play a multifaceted role in the initiation, advancement, and progression of colon cancer. Due to the complex nature of senescent cells, certain senescence-related genes likely promote while others inhibit processes contributing to the initiation, advancement, and progression of colon cancer. This intricate role was considered in the calculation of the integrated survival prediction, where the predictive effect of the senescence-related genes was assessed by accounting for the directionality of their individual gene expressions. A recent study identified three distinct senescence subtypes in colorectal cancer through integrated analysis of multiple datasets [22]. Different subtypes exhibited varying responses to chemotherapy and immunotherapy. Additionally, a senescence scoring system based on seven senescent genes showed promising prognostic value, with lower scores associated with longer disease-free survival and potential benefits from immunotherapy [22]. Moreover, a prognostic risk model consisting of six senescence-associated lncRNAs predicts survival and risk in colorectal cancer patients [21]. It has also been suggested that oxidative stress-inducing risk factors, including diabetes mellitus, may facilitate the process of tumorigenesis by promoting premature cell senescence [24]. In vitro studies suggest that senescence-related gene expression in colorectal carcinoma cells may associate with acquired resistance to chemotherapy and acquisition of a more aggressive phenotype over time [25]. The hypothesis was put forward that senescent tumor cells through their SASP protect nonsenescent tumor cells from immune attack [26]. Secretion of SASP-factors by senescent mesenchymal stem cells was proposed to promote tumor cells growth [27]. Our findings, taken together with earlier results, contribute novel insights into the intricate mechanisms underlying colon cancer pathogenesis, providing a deeper understanding of the complex senescence-related processes in the context of this disease.

While our results align with existing literature, we also identify areas of contradiction and divergence, warranting further investigation [28]. For instance, an analysis of 230 stage I-III cancers revealed that 63% exhibited high nuclear expression of the senescence marker p16^*ink4a*^, which correlated with improved survival outcomes [29]. Further, in stage IV colorectal cancer patients, an increased tumor senescence burden was shown to be associated with a significantly longer progression-free survival in response to treatment with 5-Fluorouracil/leucovorin therapy [30]. Inducing senescence in proliferating tumor cells may also confer therapeutic advantages [31].

Our study underscores the complex role of cellular senescence in driving colon cancer pathogenesis, extending findings from previous investigations. Earlier studies suggested the involvement of the SASP in promoting metastasis, as well as the contributions of senescent cells and matrix metalloproteinases (MMPs) to disease progression [32–39]. These findings highlight the multifaceted nature of senescence in influencing various aspects of colon cancer development and metastasis. Further analyses should identify specific senescence-related genes associated with disease progression, providing valuable clues for understanding the molecular pathways driving tumor growth, invasion, and metastasis.

Based on literature data, senescence emerges as a promising therapeutic target for various cancers, potentially including colon cancer [12, 15, 40–53]. By directing interventions towards senescence-related pathways, including the SASP and mechanisms for senescent cell clearance, innovative therapeutic avenues may be explored to hinder tumor progression and enhance patient outcomes [44].

Although our study offers valuable insights, it is important to acknowledge its limitations. Methodological factors, including variations in tumor stages, interactions with established prognostic biomarkers, disparities in treatment protocols, socioeconomic variables, and the analytical methods employed, have the potential to introduce biases that may impact the interpretation of our findings. Furthermore, the retrospective design of our study may restrict the applicability of our results to broader patient populations. Future research should explore the combined predictive power of senescence-related gene prediction signatures with existing biomarkers to enhance prognostic accuracy in colon cancer [54–58].

In summary, our study highlights the prognostic significance of senescence-related genes in colon cancer and provides insights into their potential roles in disease pathogenesis and progression. Moving forward, future studies should focus on validating our findings in larger patient cohorts. Future comprehensive analysis of the role of senescence-related mechanisms will likely identify novel therapeutic targets and diagnostic biomarkers, offering avenues for improving patient care and clinical outcomes.

### Supplementary Information

Below is the link to the electronic supplementary material.

Supplementary file1 (XLSX 11 KB)
